# Fisetin Protects HaCaT Human Keratinocytes from Fine Particulate Matter (PM_2.5_)-Induced Oxidative Stress and Apoptosis by Inhibiting the Endoplasmic Reticulum Stress Response

**DOI:** 10.3390/antiox10091492

**Published:** 2021-09-18

**Authors:** Ilandarage Menu Neelaka Molagoda, Mirissa Hewage Dumindu Kavinda, Yung Hyun Choi, Hyesook Lee, Chang-Hee Kang, Mi-Hwa Lee, Chang-Min Lee, Gi-Young Kim

**Affiliations:** 1 Department of Marine Life Science, Jeju National University, Jeju 63243, Korea; neelakagm2012@gmail.com (I.M.N.M.); dumindu.kaviya@gmail.com (M.H.D.K.); 2 Research Institute for Basic Sciences, Jeju National University, Jeju 63243, Korea; 3 Department of Biochemistry, College of Korean Medicine, Dong-Eui University, Busan 47227, Korea; choiyh@deu.ac.kr (Y.H.C.); 14769@deu.ac.kr (H.L.); 4 Nakdonggang National Institute of Biological Resources, Sangju 37242, Gyeongsanbuk-do, Korea; ckdgml3735@nnibr.re.kr (C.-H.K.); blume96@nnibr.re.kr (M.-H.L.); 5 Department of Molecular Microbiology and Immunology, Brown University, 185 Meeting Street, Box G-L, Providence, RI 02913, USA; Chang_Min_Lee@brown.edu

**Keywords:** fisetin, PM_2.5_, apoptosis, endoplasmic reticulum stress, reactive oxygen species

## Abstract

Fine particulate matter (PM_2.5_) originates from the combustion of coal and is found in the exhaust of fumes of diesel vehicles. PM_2.5_ readily penetrates the skin via the aryl hydrocarbon receptor, causing skin senescence, inflammatory skin diseases, DNA damage, and carcinogenesis. In this study, we investigated whether fisetin, a bioactive flavonoid, prevents PM_2.5_-induced apoptosis in HaCaT human keratinocytes. The results demonstrated that fisetin significantly downregulated PM_2.5_-induced apoptosis at concentrations below 10 μM. Fisetin strongly inhibited the production of reactive oxygen species (ROS) and the expression of pro-apoptotic proteins. The PM_2.5_-induced apoptosis was associated with the induction of the endoplasmic reticulum (ER) stress response, mediated via the protein kinase R-like ER kinase (PERK)–eukaryotic initiation factor 2α (eIF2α)–activating transcription factor 4 (ATF4)–CCAAT-enhancer-binding protein (C/EBP) homologous protein (CHOP) axis. Additionally, the cytosolic Ca^2+^ levels were markedly increased following exposure to PM_2.5_. However, fisetin inhibited the expression of ER stress-related proteins, including 78 kDa glucose-regulated protein (GRP78), phospho-eIF2α, ATF4, and CHOP, and reduced the cytosolic Ca^2+^ levels. These data suggest that fisetin inhibits PM_2.5_-induced apoptosis by inhibiting the ER stress response and production of ROS.

## 1. Introduction

The majority of fine particulate matter (PM_2.5_) originates from the combustion of coal and is found in the exhaust fumes of diesel vehicles. PM_2.5_ causes serious pathological conditions and diseases, including lung cancer, chronic inflammation of the airways, cardiovascular dysfunction, diabetes mellitus, and genotoxicity [1]. As the aerodynamic diameter of PM_2.5_ is below 2.5 µM [2], a greater fraction of these particles is deposited in the lungs from where it penetrates into the deeper tissues, causing damage to the respiratory system [3]. In addition to the lung injury, toxicological and epidemiological studies have demonstrated that the inhaled PM_2.5_ thus enters into the systemic circulation and causes severe life-threatening disorders of the nervous system [4], respiratory system [5], immune system [6], and cardiovascular system [7]. PM_2.5_ is a mixture of numerous chemicals, including metals, allergens, toxic products generated during the combustion of fossil fuels, and endotoxins. In particular, compounds such as polycyclic organic hydrocarbons readily penetrate through the skin via the aryl hydrocarbon receptor (AhR) [8], resulting in the excessive generation of reactive oxygen species (ROS). This subsequently results in skin senescence, inflammatory diseases of the skin, DNA damage, and carcinogenesis in the skin [9,10].

Piao et al. [9] demonstrated that PM_2.5_ induces oxidative stress-related endoplasmic reticulum (ER) stress in HaCaT human keratinocytes. The excessive production of ROS under adverse physiological conditions is directly linked to the induction of ER stress responses and subsequent release of Ca^2+^ from the lumen of the ER [11]. The disruption of the ER membrane decreases the protein-folding capacity, which induces the unfolded protein response (UPR) [12]. Protein kinase R-like ER kinase (PERK) contains an ER-luminal domain that monitors imbalanced protein-folding in the ER by binding to the 78-kDa glucose-regulated protein (GRP78) [13]. The phosphorylation of eukaryotic initiation factor 2α (eIF2α) by PERK inhibits the assembly of 80s ribosomes at the initiation codon of the mRNA to promote protein synthesis [14]. The eIF2α protein simultaneously interacts with the coding region of activating transcription factor 4 (ATF4), resulting in the upregulation of CCAAT-enhancer-binding protein (C/EBP) homologous protein (CHOP), which triggers the initiation of apoptotic signals [15]. The CHOP transcription factor regulates the expression of several anti-apoptotic and pro-apoptotic genes, including proteins of the Bcl-2 family [16]. The impairment of the Bcl-2:Bax ratio in the mitochondrial membrane induces the opening of the mitochondrial permeability transition (MPT) pore. This enables the release of active apoptotic substances including cytochrome *c*, which eventually results in caspase-dependent apoptosis [17].

Fisetin is a bioactive flavonoid that is found in many fruits and vegetables, including grapes, onions, and strawberries [18]. Compared to the other botanical antioxidants and polyphenols, fisetin possesses more prominent biological activities, including anti-inflammatory [19], antioxidant [20], and anti-carcinogenic [21] properties. Previous studies of Jia et al. [21] and Kang et al. [7] demonstrated that fisetin induces ER stress in pancreatic carcinoma and hepatocarcinoma, suggesting that fisetin mediates ER stress depending on the cell type. Nevertheless, the protective effect of fisetin against PM_2.5_-induced damage in keratinocytes is yet to be elucidated by targeting ER stress responses. Therefore, in this study, we investigated whether fisetin inhibits PM_2.5_-induced oxidative stress and apoptosis in HaCaT keratinocytes by inhibiting the ER stress responses.

## 2. Materials and Methods

### 2.1. Reagents and Antibodies

The National Institute of Standards and Technology (NIST, Gaithersburg, MD, USA) SRM 1650b standard diesel PM_2.5_, 3-(4,5-dimethylthiazol-2-yl)-2,5-diphenyltetrazolium bromide (MTT), and salubrinal were purchased from Sigma-Aldrich (St. Louis, MO, USA), and 2′,7′-Dichlorodihydrofluorescein diacetate (DCFDA) was purchased from Molecular Probes (Eugene, OR, USA). Primary antibodies against Bcl-2 (sc-492), Bid (sc-11423), cytochrome *c* (sc-13560), Bax (sc-7480), PARP (sc-7150), caspase-3 (sc-7272), caspase-8 (sc-81656), caspase-9 (sc-70507), GRP78 (sc-13968), CHOP (sc-575), β-actin (sc-69879), and peroxidase-labeled anti-mouse immunoglobulins (sc-16102) were purchased from Santa Cruz Biotechnology (Santa Cruz, CA, USA). Primary antibodies against eIF2α (PA5-27366), phospho (p)-eIF2α (MA5-15133), and ATF4 (PA5-19521) were purchased from Thermo Fisher Scientific (Waltham, MA, USA). Peroxidase-labeled anti-rabbit immunoglobulins (KO211708) were purchased from KOMA Biotechnology (Seoul, Korea), respectively. Dulbecco’s Modified Eagle Medium (DMEM), antibiotic mixture, fetal bovine serum (FBS), and trypsin-ethylenediaminetetraacetic acid (EDTA) solution were purchased from WELGENE (Gyeongsan, Gyeongsangbuk-do, Korea). All the other chemicals used in this study were purchased from Sigma-Aldrich.

### 2.2. Preparation of PM_2.5_ Stock Solutions

Diesel PM_2.5_ was dissolved in DMSO to prepare the stock solution (25 mg/mL). To avoid aggregation of the suspended PM_2.5_ particles, the solution was sonicated for 30 min in a water bath.

### 2.3. Cell Culture and Cell Viability

Immortalized HaCaT keratinocytes were procured from American Type Cell Culture Collection (Manassas, VA, USA) and maintained in DMEM supplemented with 10% FBS and an antibiotic mixture. The HaCaT keratinocytes were treated with fisetin (0–20 µM) for 24 h and stained with a Muse Count & Viability Kit (Luminex, Austin, TX, USA) for 5 min. The population of dead and viable cells was measured using a Muse Cell Analyzer (Luminex).

### 2.4. Annexin V Staining

HaCaT keratinocytes were treated with fisetin (0–20 μM) for 2 h prior to stimulation with 100 μg/mL PM_2.5_ for 24 h. The cells were collected and incubated with a Muse Annexin V & Dead Cell Kit (Luminex) for 30 min. The population of apoptotic cells was measured using a Muse Cell Analyzer.

### 2.5. Protein Extraction and Western Blotting

HaCaT keratinocytes were treated with fisetin (0–10 μM) for 2 h prior to exposure to 100 μg/mL PM_2.5_ for 24 h. The cells were subsequently lysed with Radioimmunoprecipitation Assay Buffer (RIPA) (iNtRON Biotechnology, Seongnam, Gyeonggi-do, Korea) with protease inhibitors (Sigma-Aldrich) and the proteins were quantified using Bio-Rad Protein Assay Reagents (Bio-Rad, Hercules, CA, USA). Western blotting was performed, and the protein expression was quantified using an ImageQuant LAS 500 Imaging System (GE Healthcare Bio-Sciences AB, Uppsala, Sweden). β-Actin was used as the loading control.

### 2.6. Caspase-3/7 Activity

HaCaT keratinocytes were treated with fisetin (0–10 μM) for 2 h prior to exposure to 100 μg/mL PM_2.5_ for 24 h. The cells were subsequently harvested and stained with a Muse Caspase-3/7 Assay Kit (Luminex), following which the cells were incubated with 7-aminoactinomycin D (7-AAD) for detecting the apoptotic cells. The population of caspase-3/7^+^ apoptotic cells was measured using a Muse Cell Analyzer.

### 2.7. Intracellular Production of ROS

HaCaT keratinocytes were treated with fisetin (0–10 μM) and subsequently stimulated with 100 μg/mL PM_2.5_ for 2 h. The population of ROS^+^ cells was measured using a Muse Oxidative Stress Kit (Luminex). In a parallel experiment, the cells were incubated with 10 μM DCFDA for 10 min and the images of the cells were captured using a CELENA S digital imaging system (Logos Biosystems, Anyang, Gyeonggi-do, Korea).

### 2.8. Cytosolic Ca^2+^ Levels

HaCaT keratinocytes were treated with 10 μM fisetin and 20 μM salubrinal in the presence or absence of 100 μg/mL PM_2.5_ for 24 h. The cells were incubated with 1 μM Fluo-4 AM for 10 min and the images of the cells were captured using a CELENA S digital imaging system.

### 2.9. ROS Staining in Zebrafish Larvae

Zebrafish (AB strain) were raised and maintained according to the standard guidelines of the Animal Care and Use Committee of Jeju National University (Jeju, Jeju Special Self-governing Province, Korea; approval no.: 2020-0051). All the experiments were performed in accordance with the ARRIVE guidelines [22]. The fertilized embryos were cultured in E3 embryo medium containing 2 mg/L methylene blue. After 3 days of fertilization, the zebrafish larvae (n = 20, per group) were pretreated with 0–400 µM fisetin for 2 h, and subsequently exposed to 50 μg/mL PM_2.5_ for 24 h. The zebrafish embryos were incubated with 20 µM DCFDA for 30 min and visualized using a CELENA S Digital Imaging System. The fluorescence intensities were determined using the ImageJ software (National Institute of Health, Bethesda, MD, USA, www.imagej.net, accessed on 26 July 2021), and the relative intensity was subsequently determined.

### 2.10. Statistical Analyses

All the data were statistically analyzed using SigmaPlot version 12.0 (Systat Software, San Jose, CA, USA, www.systatsoftware.com, accessed on 26 July 2021). The data represent the mean of the data obtained from experiments performed at least in triplicate, and are presented as the mean ± standard error of the median. The significant differences between the groups were determined using Student’s t-test and unpaired one-way analysis of variance (ANOVA) with Bonferroni correction. Statistical significance was set at *** and ^###^ *p* < 0.001, ** and ^##^ *p* < 0.01, and * and ^#^ *p* < 0.05.

## 3. Results

### 3.1. Fisetin Protects HaCaT Keratinocytes from PM_2.5_-Induced Apoptosis

**Figure 1:**
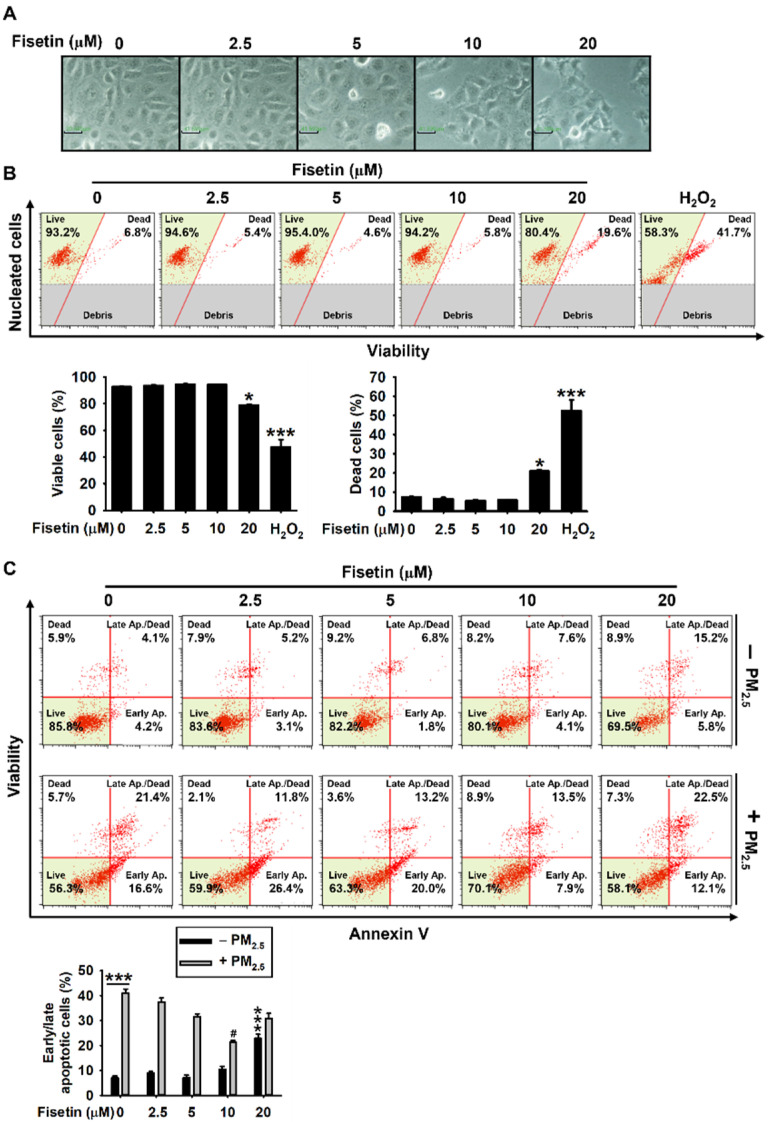
Fisetin protects HaCaT keratinocytes against PM_2.5_-induced apoptosis. The HaCaT keratinocytes were treated with fisetin (0–20 µM) for 24 h. (**A**) Morphological changes were observed by phase-contrast microscopy (10×). Scale bar = 40 µm. (**B**) Cell viability was measured using a Muse Count & Viability Kit. Graphical representation of the population of viable (*bottom left*) and dead cells (*bottom right*). (**C**) The cells were pretreated with fisetin (0–20 µM) for 2 h prior to exposure to 100 µg/mL PM_2.5_ for 24 h. Apoptosis was measured using a Muse Annexin V & Dead Cell Assay Kit. Graphical representation of the population of early/late apoptotic cells (*bottom*). *** *p* < 0.001 and * *p* < 0.05 vs. untreated cells and ^#^ *p* < 0.05 vs. PM_2.5_-treated cells.

In order to evaluate the cytotoxic effects of fisetin, HaCaT keratinocytes were initially treated with fisetin for 24 h, and the cellular morphology and viability were studied. Morphological observation revealed fisetin significantly increased the number of apoptosis-related cellular markers, including shrunken and floating cells, at the highest concentration tested herein (20 µM). However, no apoptotic morphologies were observed when the concentration of fisetin was below 10 µM (Figure 1A). Furthermore, the data obtained from flow cytometry revealed that fisetin significantly decreased the population of viable cells at a concentration of 20 µM (79.0% ± 0.7%; Figure 1B, *bottom left*) and increased the population of dead cells (21.0% ± 0.6%; Figure 1B, *bottom right*) compared to those of the untreated cells (population of viable and dead cells: 92.7% ± 0.3% and 7.3% ± 0.3%; Figure 1B). Fisetin increased the population of apoptotic cell from 7.0% ± 0.8% to 23.0% ± 1.6% at a concentration of 20 µM. However, fisetin did not increase the population of apoptotic cells at concentrations below 10 µM (Figure 1C). We also observed that exposure to PM_2.5_ significantly induced cellular apoptosis (41.0% ± 1.6%), and fisetin alleviated PM_2.5_-induced apoptosis in a concentration-dependent manner (37.5% ± 1.5%, 31.6% ± 1.1%, and 21.4 ± 0.6% at 2.5, 5, and 10 µM, respectively). Although the anti-apoptotic effect of fisetin was significant at 20 µM (30.8% ± 2.1%), the effect was lower than that at 10 µM. These results indicated that fisetin possesses no cytotoxicity at low concentrations and protects HaCaT keratinocytes against PM_2.5_-induced apoptosis.

### 3.2. Fisetin Inhibits PM_2.5_-Induced Apoptosis by Modulating the Expression of Apoptosis-Related Proteins

**Figure 2:**
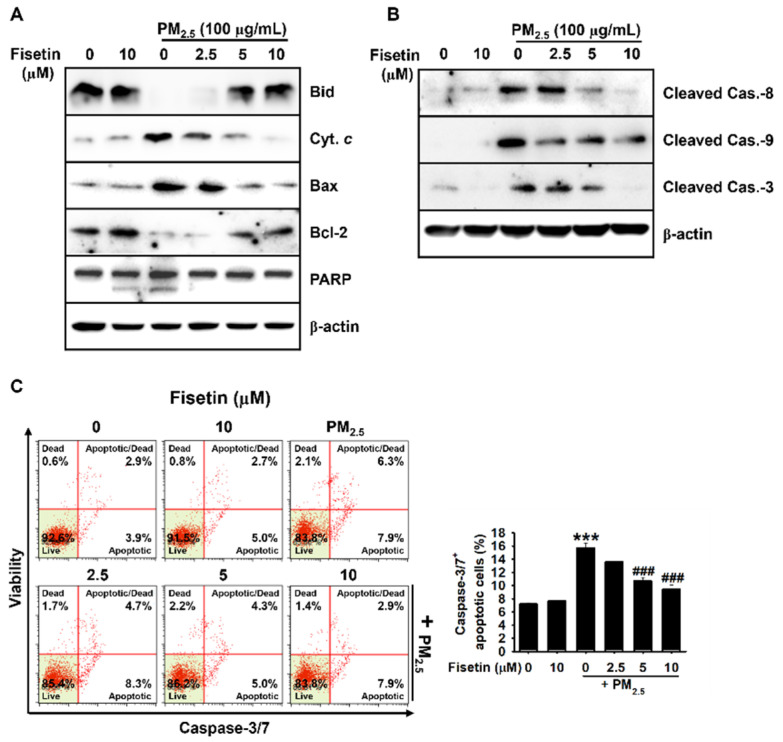
Fisetin downregulates PM_2.5_-induced pro-apoptotic activity in HaCaT keratinocytes. The HaCaT keratinocytes were treated with fisetin (0–10 µM) for 2 h prior to exposure to 100 µg/mL PM_2.5_ for 24 h. The total proteins were extracted, and Western blotting was performed for detecting the expression of (**A**) Bid, cytochrome *c* (Cyt. *c*), Bax, Bcl-2, and PARP; and (**B**) cleaved caspase (Cas.)-8, Cas.-9, and Cas.-3. β-Actin was used as the loading control. (**C**) The population of caspase-3/7^+^ apoptotic cells was measured using a Muse Caspase-3/7 Assay Kit. Graphical representation of the population of early/late apoptotic cells (*right*). *** *p* < 0.001 vs. untreated cells and ^###^ *p* < 0.001 vs. PM_2.5_-treated cells.

The members of the Bcl-2 family are considered to be the primary regulators of apoptosis following the release of mitochondria-derived apoptotic signals [23]. We therefore determined the expression of the proteins in the Bcl-2 family. As depicted in Figure 2A, fisetin effectively increased the total expression of Bid and Bcl-2 in the presence of PM_2.5_, and downregulated the expression of cytochrome *c* and Bax, which was accompanied by the cleavage of PARP (Figure 2A). As expected, exposure to PM_2.5_ significantly induced the cleavage of capase-3, caspase-8, and caspase-9, and fisetin effectively blocked their cleavage in a concentration-dependent manner (Figure 2B). In order to further validate whether fisetin inhibited caspase-induced apoptosis, the activity of caspase 3/7 and the population of caspase-3/7^+^ apoptotic cells were measured by flow cytometry (Figure 2C, *left*). The results demonstrated that exposure to PM_2.5_ significantly increased the population of caspase-3/7^+^ apoptotic cells from 6.3% ± 0.3% to 15.7% ± 0.8%, and treatment with fisetin reduced the population of caspase-3/7^+^ apoptotic cells to 13.5% ± 0.4%, 10.1% ± 0.6%, and 9.4% ± 0.7% at concentrations of 2.5, 5, and 10 µM, respectively (Figure 2C, *right*). These data indicated that fisetin protected the PM_2.5_-treated HaCaT keratinocytes from mitochondria-mediated apoptosis.

### 3.3. Fisetin Inhibits PM_2.5_-Induced Production of ROS

**Figure 3:**
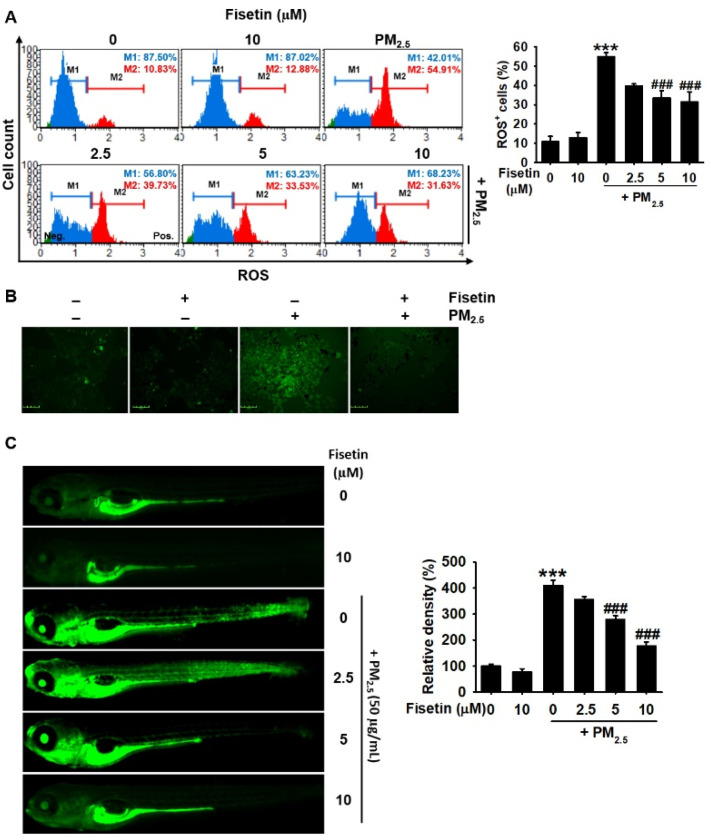
Fisetin inhibits the PM_2.5_-induced production of ROS. HaCaT keratinocytes were treated with fisetin (0–10 µM) for 2 h and subsequently exposed to 100 µg/mL PM_2.5_ for 24 h. (**A**) The cells were stained with a Muse Oxidative Stress Assay Kit and the population of ROS^+^ cells was subsequently measured. (**B**) In a parallel experiment, the cells were treated with 10 µM fisetin in the presence or absence of 100 µg/mL PM_2.5_ for 24 h. The cells were incubated with 10 μM DCFDA for 10 min and the images of the cells were captured using a CELENA S Digital Imaging System. Scale bar = 100 µm. (**C**) Zebrafish larvae at 3 days post-fertilization were pretreated with fisetin (0–400 µM) for 2 h and exposed to 50 µg/mL PM_2.5_ for 24 h. The larvae were incubated with 20 µM DCFDA for 30 min and visualized using a CELENA S Digital Imaging System. The fluorescence intensities were calculated using the ImageJ software and are expressed. *** *p* < 0.001 vs. untreated larvae and ^###^ *p* < 0.001 vs. PM_2.5_-treated larvae.

As the excessive production of ROS promotes the initiation of apoptotic pathways [24], we investigated whether fisetin alleviates the PM_2.5_-induced production of ROS in HaCaT keratinocytes and zebrafish larvae. As expected, the data obtained from flow cytometry (Figure 3A, *left*) revealed that PM_2.5_ markedly increased the population of ROS^+^ cells to 54.9% ± 2.0% compared to that of the untreated cells (10.8% ± 2.8%; Figure 3A, *right*). However, treatment with fisetin gradually inhibited the PM_2.5_-induced production of ROS in a concentration-dependent manner (39.7% ± 1.1%, 33.5% ± 3.6%, and 31.3% ± 5.2% at concentrations of 2.5, 5, and 10 µM, respectively). Fluorescence staining using DCFDA also confirmed that fisetin effectively inhibited the PM_2.5_-induced production of ROS in HaCaT keratinocytes (Figure 3B). In order to elucidate the ROS scavenging ability of fisetin in zebrafish larvae, we treated zebrafish larvae at 3 days post-fertilization with fisetin for 2 h prior to exposure to PM_2.5_ for 24 h. The data obtained by fluorescence microscopy revealed that PM_2.5_ significantly induced the production of ROS in the treated larvae, compared to that of the untreated zebrafish larvae (Figure 3C). Fisetin attenuated the PM_2.5_-induced production of ROS in a concentration-dependent manner. These results suggested that fisetin inhibited the PM_2.5_-induced production of ROS in HaCaT keratinocytes and zebrafish larvae.

### 3.4. Fisetin Inhibits PM_2.5_-Induced Apoptosis by Alleviating ER Stress

**Figure 4:**
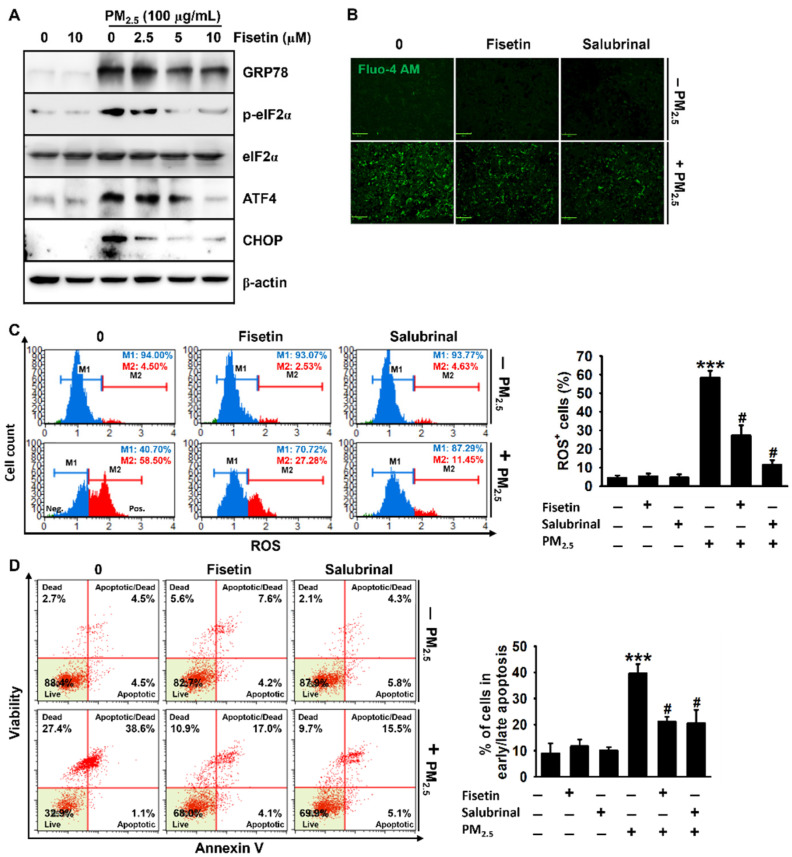
Fisetin inhibits PM_2.5_-induced apoptosis by alleviating ER stress. HaCaT keratinocytes were treated with fisetin (0–10 µM) for 2 h and subsequently exposed to 100 µg/mL PM_2.5_ for 24 h. (**A**) The total proteins were extracted, and Western blotting was performed for detecting the expression of GRP78, p-eIF2α, eIF2α, ATF4, and CHOP. β-Actin was used as the loading control. (**B**) The cells were treated with 10 µM fisetin or 20 µM salubrinal in the presence or absence of 100 µg/mL PM_2.5_ for 24 h. The cells were incubated with Ca^2+^-sensitive Fluo-4 AM for 10 min and live images were captured using a CELENA S Digital Imaging System. Scale bar = 100 µm. (**C**,**D**) In a parallel experiment, the cells were treated with 10 µM fisetin or 20 µM salubrinal in the presence or absence of 100 µg/mL PM_2.5_ for 24 h. The cells were stained with a (**C**) Muse Oxidative Stress Assay Kit and (**D**) Muse Annexin V & Dead Cell Assay Kit. *** *p* < 0.001 vs. untreated cells and ^#^ *p* < 0.05 vs. PM_2.5_-treated cells.

In order to confirm the effect of fisetin on PM_2.5_-induced ER stress, we investigated the expression of the components of the PERK–ATF4–CHOP axis following the ER stress response induced by PM_2.5_. We observed that exposure to PM_2.5_ was associated with the upregulation of ER stress marker proteins, including GRP78, p-eIF2α, ATF4, and CHOP, whereas treatment with fisetin attenuated their upregulation in a concentration-dependent manner (Figure 4A). The induction of the ER stress response by PM_2.5_ resulted in the upregulation of the cytosolic Ca^2+^ levels, and pretreatment with 10 µM fisetin markedly reduced the Ca^2+^ levels induced by exposure to PM_2.5_. Salubrinal is a non-canonical ER-stress inhibitor in which the mode of action is dependent on the inhibition of peIF2α dephosphorylation by preventing the formation of protein phosphatase-1 (PP1)/growth arrest and DNA damage-inducible protein-34 (GAD34) complex [25,26]. Therefore, here the effect of fisetin on ER-stress was compared with salubrinal and we noticed that a reduction in the Ca^2+^ levels following treatment with fisetin was comparable to that after treatment with 20 µM salubrinal (Figure 4B). Additionally, salubrinal reduced the population of ROS^+^ cells after PM_2.5_ exposure from 58.5% ± 3.6% to 11.45% ± 2.56%, and treatment with 10 µM fisetin also decreased the population of ROS^+^ cells to 27.3% ± 5.5%. Consistent with the data on ROS production, the population of apoptotic cells following PM_2.5_ exposure (39.7% ± 3.6%) was significantly reduced by treatment with salubrinal to 20.5% ± 5.2%, and fisetin also decreased the population of apoptotic cells to 21.1% ± 1.9%. These results indicated that fisetin inhibited PM_2.5_-induced apoptosis by alleviating the ER stress responses.

## 4. Discussion

The skin is the largest organ in the body and acts as an interface between the human body and the external environment. The stratum corneum regulates the passage of electrolytes, biological substances, and toxic materials through the skin; however, prolonged or repeated exposure to irritants increases the vulnerability of the skin to adverse pathological risks [27]. Accumulating evidence demonstrates that PM_2.5_ disrupts the skin barrier, which exerts adverse effects on keratinocytes and results in oxidative stress-induced apoptosis [9,28]. PM_2.5_ contains higher levels of polycyclic organic hydrocarbons that readily penetrate the skin via AhRs, and eventually promote the excessive production of ROS, ER stress, mitochondrial dysfunction, and apoptosis [8]. Plant-derived bioactive compounds, such as polyphenols, can protect against skin irritations induced by air pollutants, ROS production, and apoptosis [29]. Fisetin is a bioactive flavonoid that can scavenge ROS and chelate metal ions, both of which are involved in the generation of free radicals [18,20]. In this study, we investigated whether fisetin effectively prevents PM_2.5_-induced generation of ROS and apoptosis in HaCaT keratinocytes by inhibiting the ER stress response. In contrast, previous studies of Jia et al. demonstrated that fisetin promotes autophagy in PANC-1 pancreatic cancer cells by inducing ER-stress and mitochondrial-stress through the AMPK/mTOR signaling pathway [21]. In addition, Kang et al. also revealed that Fisetin induces apoptosis and ER stress in NCI-H460 human non-small cell lung cancer via activation of the MAPK cell signaling pathway [7], suggesting that the effect of fisetin on ER stress is depended on the cell type.

PM_2.5_ disrupts skin homeostasis by damaging the nucleic acids and proteins and disrupting cellular lipid metabolism, which subsequently induces the excessive production of ROS and apoptosis [9,10]. Intracellular Ca^2+^ also plays a crucial role in the production of ROS in response to PM_2.5_ exposure. In this regard, some polyphenolic bioactive compounds strongly rescue skin cells from PM_2.5_-induced generation of ROS and apoptosis, resulting in the growth of healthy skin [29]. In this study, we also observed that fisetin effectively decreased the levels of ROS induced by PM_2.5_ exposure in HaCaT keratinocytes. The apoptosis induced by PM_2.5_ was suppressed in fisetin-treated HaCaT keratinocytes, which was accompanied by a downregulation in caspase activity. These data indicate that fisetin protects keratinocytes against PM_2.5_-induced apoptosis. A study demonstrated that PM_2.5_ inhibits filaggrin to downregulate the aggregation of keratin bundles in keratinocytes, thereby disrupting the barrier functions of the skin [30]. These studies demonstrated that PM_2.5_ directly breaks down the proteins in the extracellular matrix (ECM), including keratin and collagen, thus causing damage to the ECM. Wu et al. [31] demonstrated that fisetin prevents the degradation of collagen and keratin in mice with UVB-induced skin damage via the antioxidant and anti-inflammatory signaling pathways. Additionally, Chamcheu et al. [32] reported that fisetin ameliorates the pathology of psoriasis by significantly increasing the content of collagen and keratin. However, it remains to be elucidated whether ECM remodeling by fisetin attenuates PM_2.5_-induced ECM damage in keratinocytes. Nevertheless, the balance between ECM remodeling and ROS-induced apoptosis may regulate the protective activity of fisetin on the skin against UVB irradiation. Further studies are necessary for evaluating whether fisetin protects human skin cells from severe PM_2.5_-induced damage.

The prolonged exposure of PM_2.5_ activates the ER stress response by mobilizing Ca^2+^ from the lumen of the ER, leading to the activation of the UPR-dependent PERK pathway and disruption of cellular homeostasis [33]. A study demonstrated that the pharmacological inhibition of ER Ca^2+^-ATPase pumps by thapsigargin potentially depletes the Ca^2+^ levels in the ER and increases the accumulation of cytosolic Ca^2+^, causing cellular apoptosis [34]. In this study, we observed that PM_2.5_ (100 μg/mL) increased the levels of cytosolic Ca^2+^, which was associated with the upregulation of GRP78, causing ROS-induced apoptosis. Additionally, the disruption of ER homeostasis by PM_2.5_ is associated with the loss of mitochondrial membrane potential, and subsequent disruption of the balance between the expression of anti-apoptotic Bcl-2 proteins and pro-apoptotic Bax, causing caspase-mediated apoptosis. In this study, we observed that fisetin effectively inhibited ER stress and consequently prevented the excessive accumulation of cytosolic Ca^2+^. Pio et al. [9] also reported that PM_2.5_ (100 μg/mL) triggers the production of ROS, increases mitochondrial damage, and induces ER stress. These data indicate that fisetin may be a potent bioactive compound against PM_2.5_-induced apoptosis in keratinocytes. On the contrary, high concentrations of PM_2.5_ (1 mg/mL) inhibits ER stress and activates the inflammatory response in HaCaT keratinocytes [35], indicating that high concentrations of PM_2.5_ give rise to ER stress-mediated chronic skin diseases. Nevertheless, further studies are necessary for investigating whether fisetin is able to protect against chronic skin diseases caused by high concentrations of PM_2.5_.

## 5. Conclusions

**Figure 5:**
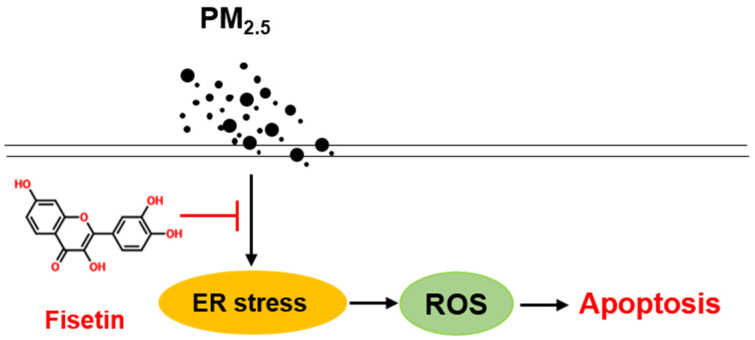
Fisetin prevents PM_2.5_-induced apoptosis in HaCaT keratinocytes by inhibiting endoplasmic reticulum (ER) stress-mediated reactive oxygen species (ROS) production.

Fisetin potentially inhibited the PM_2.5_-induced ER stress response by downregulating the PERK–eIF2α–ATF4 pathway and reducing the production of ROS and Ca^2+^ accumulation (Figure 5). The study further demonstrated that fisetin inhibited caspase activity and the expression of pro-apoptotic proteins to attenuate PM_2.5_-induced apoptosis. In conclusion, the results of this study support that fisetin potentially protects keratinocytes against PM_2.5_-induced skin damage.

## Data Availability

The data presented in this study are available on reasonable request from the corresponding author. The data are not publicly available due to privacy restrictions.
